# Exploring paths to participation and non-participation in physical exercise among Swedish adolescents

**DOI:** 10.3389/fpubh.2026.1723898

**Published:** 2026-02-03

**Authors:** Jennifer Gothilander, Edward J. Miech, Lena Almqvist, Johanna Fritz, Camilla Eriksson

**Affiliations:** 1 School of Health, Care and Social Welfare, Mälardalen University, Västerås, Sweden; 2 Indiana University School of Medicine, Indianapolis, IN, United States

**Keywords:** cluster analysis, coincidence analysis, disability, gender, sport

## Abstract

**Background:**

Physical exercise (PE) is important for health. Girls are reported to participate less compared to boys. Multiple factors influence participation and non-participation, including neighborhood, socioeconomic status, social support, and disability. Factors may combine and form paths to participation or non-participation, yet these paths are unknown. To our knowledge, this is the first study to combine cluster analysis with a configurational comparative method to explore paths to participation and non-participation in PE among adolescents.

**Methods:**

Data from 178 Swedish 15–18-year-olds revealed two exercise-related clusters: *Not exercising* and *Sporting & Exercising*. Girls and boys in these clusters were analyzed separately by coincidence analysis to identify paths leading to membership in each cluster. The initial analysis included 41 questions aggregated into 24 variables.

**Results:**

Not having quick access to pocket money is a path by itself to *Not exercising* among girls and part of the path to *Sporting & Exercising*. The paths to *Not exercising* are more complex for boys. Participation in adult-led activities differs between boys in the *Not exercising* and *Sporting & Exercising* clusters. Having a disability is only a difference-making factor among boys when combined with “not avoiding anyone in the neighborhood” and “sometimes meeting friends in person.”

**Conclusion:**

Multiple distinct paths lead to *Sporting & Exercising* and *Not exercising,* and these differ between girls and boys. Paths to exercising and non-exercising can be multi-factorial in nature, where several factors must be jointly present to explain membership in a particular exercise-related cluster. Future research may wish to adopt a similar configurational approach to explore other high-priority activities in which adolescents do or do not participate to improve interventions and policies aimed at increasing adolescent participation in PE.

## Introduction

Physical exercise (PE) is a subcategory of physical activity (PA) that is planned, structured, and repetitive, with the objective of maintaining or improving physical fitness (1, 2). PE can be done in a sports club or other contexts and contribute to adolescents’ moderate to vigorous PA (MVPA) levels (3–5). Due to the well-documented health benefits of participating in MVPA, such as PE (6–9), there is a need to understand what combination of factors contributes to adolescents’ non-participation in PE. A previous cluster analysis of PE and screen time among Swedish adolescents identified a heterogeneous cluster of non-participation in PE (10). To improve interventions and policies aimed at increasing PE participation in this age group, it is important to examine the multiple underlying factors to explain membership in these discrete exercise-related clusters. In this paper, we use an exploratory, case-oriented approach to identify bundles of factors that are difference-makers in terms of explaining participation and non-participation in PE by boys and girls.

**Figure 1:**
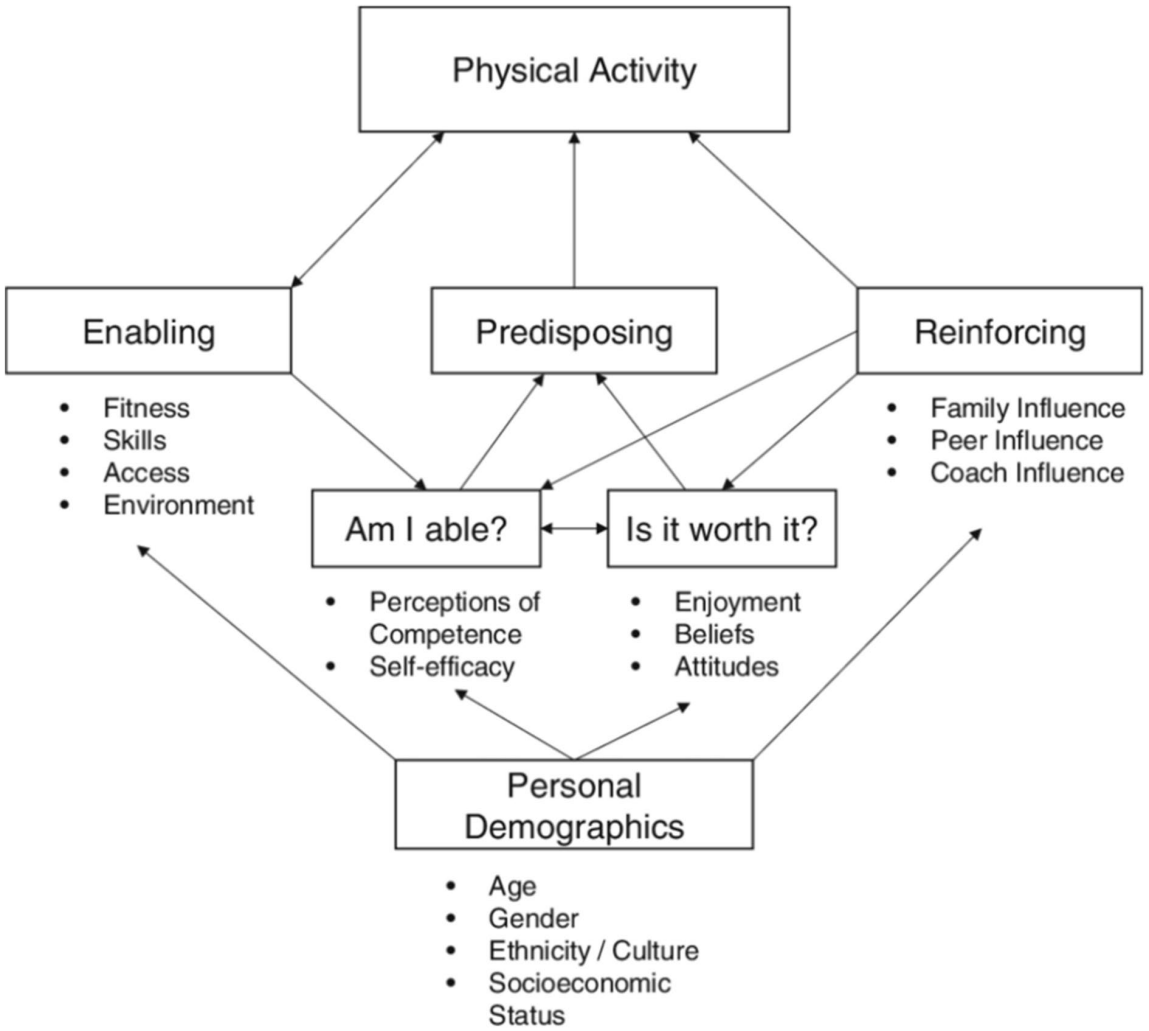
Welk’s youth physical activity promotion model (14).

It is well established that more boys than girls participate in PE (11–13). According to Welk’s Youth Physical Activity Promotion Model (14), gender is one of the factors influencing participation (Figure 1). Several scholars have also described how gender stereotypes and the masculine tradition in sports negatively influence girls’ participation in sports and PE (15–19). In addition, Welk’s model highlights that factors beyond gender, such as socioeconomic status (SES), friends or disability, also likely influence participation.

Factors such as neighborhood characteristics and indicators of SES, such as income or education, have been associated with non-participation in PE (13, 20–23). Adolescents living in neighborhoods with high availability and accessibility of parks and recreational areas are more likely to participate compared to those in neighborhoods with low availability and accessibility (13, 20, 24). Conversely, concerns about crime predict lower participation (21, 24), whereas feeling safe in one’s neighborhood increases the odds for PE (25).

Living in a deprived neighborhood is often associated with low SES (26, 27). The relationship between SES and participation in PE appears confirmed (11, 13, 28, 29). However, Rittsteiger et al. found that the effect of SES on PE decreased when controlling for opportunities for PE and social support (13). This indicates that living in a deprived neighborhood or having a low SES may each constitute distinct paths to non-participation in PE or operate together. However, the combination of these factors and social support needs further research.

Social support can come from parents, teachers, or peers. While parental influence on adolescents’ behavior may decrease with age, peer influence often increases (30). Peer support has been positively associated with adolescents’ participation in PE (31, 32). Among adolescents who spend the most time on PE, 88% report that most or almost all of their friends also participate in PE during leisure time (28). It remains unknown whether a lack of participating friends alone, or a configuration including this factor, neighborhood, SES, and parental support, creates paths to non-participation in PE, and whether such paths differ for girls and boys.

In addition, adolescents with disabilities participate less in PE compared to adolescents without disabilities (33–35). Still, previous findings showed no significant overrepresentation of adolescents with various physical and mental disabilities in clusters of non-participation in PE, including allergies, asthma, dyslexia, mobility impairment, Attention-Deficit Hyperactivity Disorder, Autism Spectrum Disorder, hearing impairment, and vision impairment (10). Thus, it remains unknown if and how disability configures with other factors to form a path to non-participation in PE.

In summary, several factors appear related to participation and non-participation in PE, yet it remains unknown which specific paths they form and whether these differ for girls and boys. In line with calls for a more holistic approach to research of adolescents’ health-related behaviors (36–38) this study aims to explore paths that lead to participation and non-participation in PE among girls and boys, and to examine whether disability is included in these paths.

## Materials and methods

The study uses a cross-sectional design and an exploratory, case-based data analysis approach. The study has been approved by the Swedish Ethical Review Authority (Dnr 2021-04388) and is conducted in accordance with the Declaration of Helsinki.

### Participants

The secondary data are from Statistics Sweden’s survey of Swedish adolescents’ living habits (Barn-ULF). Participants in Barn-ULF were recruited through the Survey of Swedish Living Conditions and Statistics on Income and Living Conditions (39). In these surveys, adults with children aged 12–18 years were asked if their child would like to participate in the Barn-ULF survey. If the adult approved, the adolescent was invited. In the year 2018, 1,149 adolescents aged 12–18 years were invited, and 607 agreed to participate (40). Data on gender and disability were provided by the adolescent’s caregiver.

Data from Barn-ULF were previously used to identify clusters based on participation in PE, sports, and screen time (10). This secondary analysis focused on data from 304 adolescents aged 15–18 years. The outcomes of interest in the current study are to belong to the cluster *Sporting & Exercising* or the cluster *Not exercising*. Of the 304 adolescents, 178 belonged to these clusters and are included in the analysis.

The *Sporting & Exercising* adolescents are characterized by participating in organized sports and PE, whereas the *Not exercising* adolescents are characterized by not participating in PE (see Table 1). The 178 adolescents in these two clusters have similar screen time, a mean age of 16.24 years, and 22% have a disability (Table 1).

**Table 1:** Participants’ characteristics.

Participation cluster	Adolescents, n (%)	Age, mean (sd)	Girls, n (%)	Have a disability, n (%)	Participate in physical exercise (PE), n (%)	Participate in sports, n (%)	Hours per weekday playing video games^#^, mean (sd)	Hours per weekday watching TV & movies^#^, mean (sd)
Not exercising	69 (38.8)	16.25 (1.18)	45 (65.2)	17 (24.6)	0 (0)	31 (44.9)	1.51 (0.63)	1.9 (0.65)
Sporting & Exercising	109 (61.2)	16.24 (1.05)	50 (45.9)	22 (20.2)	109 (100)	109 (100)	1.51 (0.54)	1.72 (0.45)
**All clusters**	**178 (100%)**	**16.24 (1.1)**	**95 (53.4)**	**39 (21.9)**	**109 (61.2)**	**140 (78.7)**	**1.51 (0.57)**	**1.79 (0.54)**

#scale of 1 to 4 (1 = Not at all, 2 = 1–2 h, 3 = 3–4 h, 4 = 5 h or more). All clusters presents mean and proportions for the full sample of 178 adolescents.

### Configurational comparative methods and coincidence analysis

Coincidence analysis (CNA) is a configurational comparative method (CCM). CCMs use Boolean algebra and regulatory theory to identify configurations (combinations of factor values) that lead to outcomes (41) and configurations are described as paths. In CCMs, model output is at the level of “conditions,” where conditions refer to factor values (i.e., when factors take on specific values). CCMs are well-suited for studying complex outcomes (42).

Unlike variable-oriented approaches that assume a linear relationship between variables and outcomes, such as ‘the more of X, the more of Y’, a CCM instead assumes a necessary or sufficient relationship, such as ‘if X, then Y’ (43). Thus, a CCM aims not to identify effect sizes of all possible variables that influence an outcome but to identify the minimally necessary conditions (conditions always present with the outcome) and sufficient (conditions co-occurring with the outcome). CCMs allow for model equifinality, meaning that there may be multiple paths that lead to the same outcome, and conjunctivity, denoting that a condition may only become a difference-maker if it is in a configuration with another condition (42). CNA uses a bottom-up approach with a rigorous minimization algorithm (44, 45) to ensure that any redundant, superfluous, or extraneous conditions are removed from models and only key difference-makers remain.

CNA is implemented in the R package “cna” (45). Consistency and coverage parameters are measures of model fit in CNA and take a value between 0 and 1. Consistency measures the reliability of models. For example, if the consistency threshold is set to 0.75, the CNA model will correctly identify cases with the outcome of interest 75% of the time or higher. Coverage measures explanatory breadth (45). For example, a model with a coverage of 0.3 accounts for 30% of all cases with the outcome present. Together, consistency and coverage ensure that CNA models account for a substantial number of cases with the outcome with high reliability.

### Data analyses

All data were prepared and analyzed in R version 4.4.0 (46) and R Studio (47) using the R packages dplyr (48) and cna (45).

Although non-participation in PE is the primary public health concern motivating this study, a CNA was conducted for both outcomes, participation and non-participation, to identify distinct and potentially asymmetric pathways to each cluster. The two outcomes *Sporting & Exercising* and *Not exercising* were analyzed separately.

As membership in the cluster *Not exercising* is particularly associated with being a girl (10), analyses of boys and girls were conducted separately in the primary analysis. To reduce fragmentation, possible difference-making factors were aggregated and calibrated before analyses. Factors, aggregation, calibration, and descriptive statistics are presented in Supplementary Table 2, and the rationale for factor selection, aggregation and calibration is presented in Supplementary Table 1. The exploratory and iterative analysis phase included 41 questions, which had been aggregated into 24 variables. The msc function in the cna package was used to reduce the number of conditions included in the CNA. The CNA was conducted with initial consistency and coverage thresholds set to 0.75, and the coverage threshold was lowered until sufficiency models were identified (0.5 for girls and 0.6 for boys). In a secondary analysis, an exploratory data analysis using the msc routine was also conducted separately on a combined dataset including girls and boys together to allow for additional insights to emerge and reduce the risk of reinforcing binary assumptions about gender.

## Results

The exploratory data analysis identified a subset of nine candidate factors to carry forward into the modeling phase: *Avoiding people in the neighborhood, Meeting friends in person, Having a disability*, *Having quick access to pocket money*, *Meeting friends online, Having a migrant background,* and *Following the news*, *Talking to a friend when being anxious or worried* and *Doing other adult-led activities*.

One girl and four boys were excluded due to missing data in one of the nine candidate factors. Thus, the sample comprised 94 girls and 79 boys (Table 2).

**Table 2:** Cases included in this study.

Gender	Not exercising, *n* (%)	Sporting and exercising, *n* (%)
Girls	44 (46.8)	50 (53.2)
Boys	24 (30.4)	55 (69.6)
Both girls and boys	**68 (100)**	**105 (100)**

All girls and boys presents n (%) in all cases included in this study.

**Figure 2:**
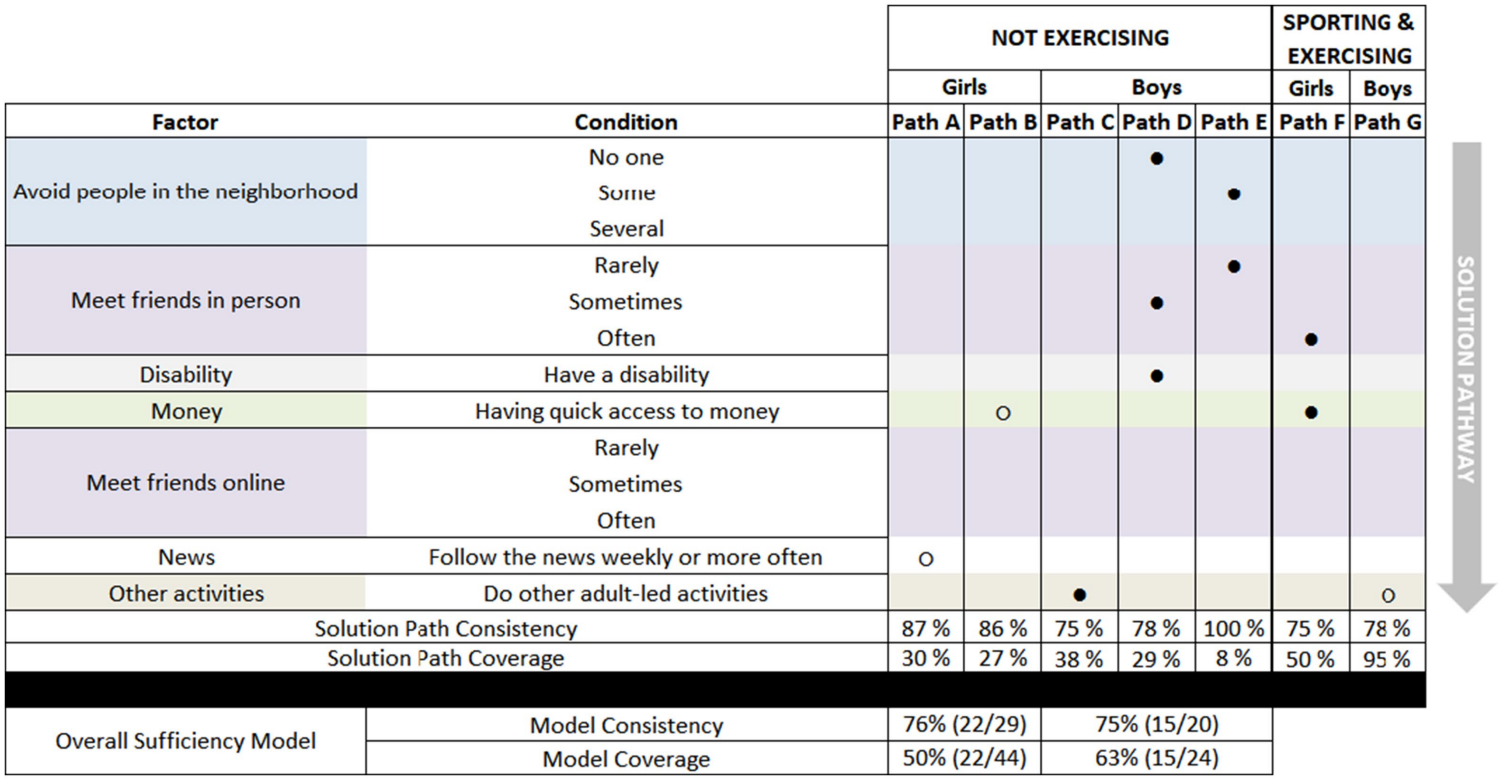
CNA models for girls and boys belonging to the clusters *not exercising* and s*porting & exercising.* Paths are “AND” configurations of conditions. Models are “OR”-configurations of paths. ● indicates the presence of the condition. ○ indicates the absence of the condition.

The configurations of specific factor values (conditions), which are described as paths, to belonging to the clusters *Not exercising* and *Sporting & Exercising* are illustrated in Figure 2.

### Girls

The sufficiency model for girls belonging to the cluster named *Not exercising* ultimately includes two paths A and B (Figure 2). The model demonstrates the concept of equifinality, i.e., that several paths may lead to the same outcome.

With the single conditions of not following the news or not having quick access to pocket money, Path A and Path B show that these are sufficient conditions in themselves for belonging to the cluster of not participating in PE (named *Not exercising*). Of all 94 girls included in the analyses, 14 do not have access to pocket money, and 15 do not follow the news weekly or more often. Of the 14 girls who do not have access to pocket money, 12 belong to the *Not exercising* cluster, translating into 86% consistency for Path A. Path B includes 13 of the 15 girls who do not follow the news weekly or more often (87% consistency). Not having quick access to pocket money (Path A) accounted for 12 of the 44 girls in the cluster *Not exercising* (50% coverage), and Path B accounted for 13 of the 44 girls (30% coverage). Of the 29 girls identified by the overall sufficiency model of Path A and Path B, 22 are in the *Not exercising* cluster, and the model accounts for 22 of the 44 girls.

The sufficiency model for girls belonging to the *Sporting & Exercising* pattern includes one path (Figure 2, Path F) with two conditions: Having quick access to pocket money and often meeting friends in person. Path F accounts for 25 of the 50 girls in the pattern and correctly identified 25 of the 33 girls with this path as belonging to the *Sporting & Exercising* cluster. The consistency and coverage for the solution of Path F are identical to the overall sufficiency model consistency and coverage and are reported only once.

### Boys

The sufficiency model for boys for belonging to the *Not exercising* cluster includes six conditions across three paths (Figure 2, Path C-E). The model demonstrates the concept of conjunctivity, i.e., conditions may only be relevant in combination with other factors.

Path C includes the single condition of doing other adult-led activities. The conditions in Path D are not avoiding anyone in the neighborhood, and sometimes meeting friends in person, and having a disability. Path E includes avoiding some people in the neighborhood and rarely meeting friends in person. Together, these three paths construct the overall sufficiency model for boys belonging to *Not exercising,* accounts for 15 of the 24 boys in the pattern (63% coverage), and correctly identifies 15 of the 20 boys in the cluster (75% consistency).

For boys belonging to the cluster *Sporting & Exercising*, there is one path with the condition of not doing other adult-led activities (Figure 2, Path G). This condition is found in 67 of the 79 boys, of which 52 belong to *Sporting & Exercising* (78% consistency). The path accounts for 52 of the 55 cases (95% coverage). The consistency and coverage for the solution of Path G are identical to the overall sufficiency model consistency and coverage and are reported only once.

### Girls and boys

There were no conditions that were common across the sufficiency models for boys and girls. Rather, as Figure 2 shows, the conditions in the models were completely different. In addition, in the secondary exploratory analysis of a combined dataset with girls and boys together, the same gender-specific paths for girls and boys emerged on their own as top-scoring paths to non-participation in PE, underscoring their role and importance.

## Discussion

To our knowledge, this is the first study to combine cluster analysis with a configurational comparative method to explore paths to participation and non-participation in PE among girls and boys. The first main finding is that multiple distinct paths lead to *Not exercising,* and these differ between girls and boys. For girls, having access to pocket money was a key difference-maker: by itself, not having access to pocket money was sufficient for belonging to the *Not exercising* cluster, whereas having access was a difference-maker for belonging to the *Sporting & Exercising* cluster when combined with meeting with friends often in person. For boys, the paths were more complex. Still, participation in other adult-led activities was a crucial difference-maker distinguishing membership in the *Not exercising* and *Sporting & Exercising* cluster among boys. The second main finding is that disability appeared as a condition only among boys, and only in combination with not avoiding anyone in the neighborhood, and sometimes meeting friends in person.

This study confirms the importance of SES for girls’ participation in PE. In our study, SES was operationalized as quick access to pocket money. Previous studies have found that girls with low SES participate in organized sports less often than boys or girls with higher SES (12, 13, 36, 49, 50), whereas a systematic review found no effect of SES on PE participation (51). Our findings thus contrast with the review. These differences may reflect cultural and international differences in PE participation and socioeconomic conditions, as well as differences in measurement.

The second path for girls, which included the condition of not following the news, may also reflect low SES. Growing up outside a ‘white-collar-home’ (52) or living in a deprived neighborhood (53) decreases adults’ odds of consuming news. A possible explanation for SES appearing as a condition only among girls is that boys and girls from low SES backgrounds face different barriers and facilitators for participation (54). In addition, gender stereotypes may affect parents’ willingness to invest in girls’ PE participation (55). Thereby, low SES, which requires more difficult prioritizations, may affect girls differently than boys.

In contrast to the two paths identified for girls, the boys’ paths are more complex, including six conditions. The boys’ model comprises three distinct paths, each potentially representing subgroups that nonetheless share the same pattern of non-participation in PE. These results may help explain the high heterogeneity observed in this pattern (10), illustrated by equifinality and conjunctivity. The equifinality highlights that multiple paths lead to the same pattern, and the conjunctivity of several conditions, which individually are not sufficient for the pattern but become difference-makers when they are combined with other conditions.

A key difference-maker for boys in explaining membership in the *Not exercising* or *Sporting & Exercising* cluster was participating in other adult-led activities. This was also the largest contributor to the overall model. In Sweden, many adolescents attend Swedish youth recreation centers, which offer adult-organized activities such as dance, excursions, leadership training, and assistance with homework. However, the primary motive for coming to these centers is often to meet friends (56). As our findings show that participation in other adult-led activities and meeting friends appeared in separate paths, youth centers may indicate two subgroups among the boys. Parents may also encourage organized, adult-led activities to keep boys away from potentially harmful or risky situations (29, 55). While organized activities can be meaningful and beneficial for health, they may not help boys achieve the recommended levels of MVPA.

Having a disability was not identified as a relevant condition for girls’ cluster belonging, but it appeared in one path for boys. Among boys in the *Not exercising* cluster, 38% had a disability compared to 25% of all boys in the study. However, the results show that disability was only relevant when combined with other conditions, suggesting that having a disability alone is insufficient to explain non-participation in PE.

Together, these results provide additional support for the interplay of factors like gender, SES, disability, and friends in the conceptual framework outlined in Welk’s Youth Physical Activity Promotion Model. By identifying how specific conditions become difference-makers, these findings also offer a new level of granularity, nuance, and detail that illustrate how particular factors within Welk’s model work together to account for participation and non-participation in PE in a real-world setting.

### Implications

This study has several implications for future research and policy. Future research should examine in particular which activities adolescents in the *Not Exercising* cluster participate in. For girls, shopping and social media are possible activities (57, 58), while for boys, it is important to explore the types of other organized activities in which they participate. Belanger et al. found that even when adolescents share the same participation pattern, their experiences and trajectories vary, underscoring the need for approaches tailored to subgroups (59). A deeper understanding of Swedish adolescents’ preferences and experiences, as well as the acceptability of various activities, is therefore needed. Addressing inequalities in PE due to SES or gender stereotypes requires action at the societal level rather than individual level. In 2025, the Swedish government launched “Fritidskortet” (translates to “leisure-time card”), an allowance intended to support children’s and adolescents’ participation in organized activities such as sports or culture (60). Still, as adolescents commonly transition from organized sports to PE as they age (61–64), and Fritidskortet is only provided until age 16, its effect on overall PA in older adolescents may be limited. While Fritidskortet aims to reduce SES-related inequalities by granting higher financial support to families receiving housing allowances, it may not address gender inequalities in participation. We encourage researchers to evaluate the effects of Fritidskortet on MVPA among adolescent girls and boys.

### Limitations and strengths

In this study, we used a measure of adolescents’ ability to quickly acquire a smaller amount of pocket money (equivalent to approximately 20 euros or 20 USD) to indicate SES. We are aware that a ‘material paradox’ of adolescents in families with lower social class receiving more pocket money compared to adolescents with higher social class has been reported (65). Still, we included parental occupation as a social class marker in the exploratory phase, and it did not appear as a difference-making factor.

Non-participation in PE is often studied combined with screen activities, video gaming, and watching TV or movies. Table 1 shows that screen time did not vary substantially between adolescents in the clusters. Unfortunately, the dataset does not include measures on social media, which could have been relevant to include, especially for girls (58, 66). Still, meeting friends online was included in the analyses. In addition, the dataset does not contain more information on the adult-led activities in which the adolescents participate.

This study examined a relatively small number of cases. While the minimized, redundancy-free solutions generated using the CNA approach identified key difference-makers, these findings may not necessarily generalize to adolescent populations substantially different from the subjects in this study. We also acknowledge the potential to mislabel adolescents’ gender identity in this study, as caregivers provided data on adolescents’ gender. However, we see the risk of mislabeling as small, and it should therefore have a limited impact on the results.

While the final CNA sufficiency models identify difference-making pathways for half or more of girls and boys in the two exercise-related clusters, a number of adolescents still remained unaccounted for by these sufficiency models, indicating that other important factors not present in the dataset were needed to fully explain membership in the two clusters.

## Conclusion

This study applied a unique combination of cluster analysis and a configurational comparative method to explore multiple factors for participation and non-participation in PE among girls and boys with and without disabilities. The analysis indicates that SES is a difference-maker for girls’ non-participation in PE. It remains unclear, though, which leisure-time activities these girls participate in. Future studies should investigate the types of activities that replace PE for this group. For boys, the findings display more complex paths to participation and non-participation in PE, with participation in other, adult-led activities serving as a key difference-maker. There is a need for research on what these activities are and if they contribute to MVPA. Finally, disability emerged as a difference-maker only among boys, and only in combination with not avoiding anyone in the neighborhood, and sometimes meeting friends in person. This suggests that having a disability alone is not sufficient to predict membership in a non-participation pattern of PE. Future research may wish to adopt a similar configurational approach to explore other high-priority activities in which adolescents do or do not participate to improve interventions and policies aimed at increasing adolescent participation in PE.

## Data Availability

The data analyzed in this study is subject to the following licenses/restrictions: the data that support the findings of this study are available from Statistics Sweden but restrictions apply to the availability of these data, which were used under license for the current study, and so are not publicly available. Data are however available from the authors upon reasonable request and with permission of Statistics Sweden. Requests to access these datasets should be directed to Statistics Sweden, scb.se.
