# Supplementary material for: Exploring paths to participation and non-participation in physical exercise among Swedish adolescents
Source: Front Public Health. 2026 Feb 3;14:1723898. doi: 10.3389/fpubh.2026.1723898 (PMC12909470; doi:10.3389/fpubh.2026.1723898)
Supplement: Supplementary file 1 [file Table_1.docx]

| **Variable** | **Question** | **Aggregation and calibration rationale** |
| --- | --- | --- |
| **Neighborhood** | *Are there kids/adolescents you want to avoid in your neighborhood?*  *Are there adults you want to avoid in your neighborhood?*  *How safe do you feel in your neighborhood during the night?*  *How safe do you feel in your neighborhood during the day?*  *How safe do you feel on your way to school?* | These two questions are similar in the avoidance of people in the neighborhood. Avoiding people could be interpreted as feeling tense and nervous in the neighborhood. We did not consider it important to distinguish between avoiding kids/adolescents or adults, but to instead focus on the consequences avoidance may have on non-participation. Instead of choosing one of these variables to include, we aggregated them into one multi-value scale to reduce fragmentation. We consider the cumulative effect of avoiding both youths and adults worse than only avoiding one of these groups.  The three questions about feeling safe in the neighborhood vary by the time of the day. The three variables are more informative for non-participation as an aggregated multi-value scale than the three individual variables. We assume that the cumulative effect of feeling somewhat unsafe all the time is qualitatively different from feeling unsafe at one time of the day. Therefore, we calibrated feeling unsafe at only one time of the day into feeling somewhat unsafe, and feeling somewhat unsafe at several times of the day into feeling unsafe. |
|  |  |  |
| **Born in Sweden versus abroad** | Country of birth | The data on this multi-value variable is gathered from a national register of the Swedish population and was connected to the cases by Statistics Sweden before we were given the dataset. To reduce fragmentation, we calibrated the variable into ‘born in Sweden’ or ‘born abroad’. While acknowledging the possible impact that parents' migrant background may have on the adolescents' identity, culture etc., we primarily focus on the adolescents’ migration background. |
|  |  |  |
| **Socioeconomic status** | Parents’ occupational class  If you suddenly need 200 SEK for tomorrow (for example, to go to the movies), could you afford it yourself?  *During the past six months, has it happened that you… have not been able to purchase something you wanted and that many others in your age has, because it was too expensive?*  … *have not been able to join your friends to something because it was too expensive?* | Socioeconomic status (SES) is commonly measured through education and income (1), and occupational class is related to both education and income (2). Parents’ occupational class was used as an indicator of SES. The data set contained 14 categories for parents’ occupations, which we calibrated according to Statistic Sweden’s guidelines (3). This variable likely predetermines the other SES variables described below, and all three variables were included in the exploratory phase to identify which ones to include in the cna.  The variable of having quick access to money indicates the adolescents’ own financial resources and is a complementary variable to their parents’ occupational class. As an example of how having access to money could affect participation in physical exercise, there are entry fees to facilities like swimming or climbing halls. Access to money for spontaneous visits would be a difference-maker for participating in such exercise activities.  Lacking money to do what peers do or buy what peers have was included as an indicator of adolescents’ relative financial inequality. This variable likely depends on the parents’ occupational class and may interact with the adolescent’s financial resources. Still, it is an indicator of SES in adolescents’ social contexts. This way, having quick access to 200 SEK, as in the previous variable, may not be enough to join your friends for more expensive activities, or it may be much more money than your friends have access to. Therefore, financial inequality may be a difference-maker for non-participation in physical exercise with friends. The two multi-value questions of financial inequality were aggregated to a multi-value scale of 0-4 and calibrated into a binary variable of having or not having experienced financial inequality. |
|  |  |  |
| **School grade** | What grade are you in? | In Sweden, junior secondary school is compulsory and includes the same school subjects for all students. In contrast, upper secondary school is focused on a chosen subject (such as media, IT, finance, etc). Vocational and upper secondary school are not separated, yet some upper secondary school subjects focus on professions such as car mechanic, truck driver, hairdresser, painter, etc.  Usually, adolescents are 15-16 years old when they graduate junior secondary school and are eligible to begin the three-year-long upper secondary school. The adolescent must be eligible for upper secondary school, and admission to schools and subjects depends on the applicants’ summarized junior secondary school course grades. In 2022, 85% of Swedish adolescents graduated from junior secondary school with eligibility for upper secondary school admission, and 74% graduated from upper secondary school (4).  Being an upper secondary school student may decrease participation in physical exercise (5). The school was included in the first phase of the analysis to explore if being a junior or upper secondary school student is a difference-maker for non-participation. The variable was calibrated into a binary junor or upper secondary school variable. |
|  |  |  |
| **Relationship with parents** | *How do you get along with your mom?*  *Do you talk to your mom if you’re anxious or worried?*  *How do you get along with your dad?*  *Do you talk to your dad if you’re anxious or worried?* | The four questions are aggregated and calibrated into two variables: the relationship with the mother and the relationship with the father.  For each parent, the first question of how they get along is multi-value, whereas whether the adolescents talk to their parent when they are anxious or worried is binary. While both questions are important, we assume adolescents do not talk about their emotions with a parent they do not get along with. Due to the assumed dependency in these questions, we aggregated them and considered the binary question as an weight in a multi-value scale. For example, getting along pretty well (=3) with the mother will stay as pretty well (=3) if the adolescent does not talk to her when they are anxious. However, if they get along pretty well (=3) and the adolescent does talk to the mother when they are anxious, we add that value so the adolescent is rated as getting along very well (=4) with their mother.  The aggregated variable for the relationship with the parent has a multi-value scale (=0-5), further calibrated to decrease fragmentation. With the highest value (=5) the adolescents get along very well and talk to their parent if they are anxious. The lowest value includes the values 0-2, indicating that they either get along poorly with the parent but talk to them if they are anxious or get along neither poorly nor well with the parent but do not talk to them when they are anxious. We argue that even if an adolescent talk to their parent when they are anxious, the statement is that the relationship is pretty poor, and talking to their parent cannot fully compensate that rating. In contrast, getting along neither poorly nor well and talking to the parent when anxious is included in having an decent relationship with the parent (values 3-4). Talking to the parent when anxious thereby move the ‘neither poorly or well’-rating into a decent relationship, but it does not move a ‘pretty poor’ into a decent relationship. |
| **Strict parents** | How strict are your parents with knowing where you are if you’re not at home? | Strict parents may limit adolescents’ possibilities for participation in physical exercise (6). In addition, it may indicate parents want to control where adolescents are allowed to be and may indicate parents' perspective on neighborhood safety. Positive and negative aspects could be included in the question, and it should be noted that this question assesses the adolescents’ perspective on how strict their parents are. |
|  |  |  |
| **Friendships** | *How often do you have friends over?*  *How often are you at a friend’s place?*  *How often do you meet friends elsewhere than someone’s home?*  *How often do you meet friends online?*  *How often do you talk on the phone or text message with friends?*  If you worry about something, do you talk to your partner/friend? | Five multi-value questions on meeting friends were aggregated into two multi-variables, which were further calibrated. We do not consider any of these five questions possible to individually exclude as they are probably all important for the variable of friendship. Therefore, they may be more informative as aggregated variables of how frequently the adolescent meet with friends. We assume that meeting friends more often indicates a more actively engaging and emotionally closer relationship than meeting them rarely. Higher values therefore indicate that adolescents are more emotionally involved in the friendship. The values in the aggregated variable were calibrated into a three-value scale to maintain a scale yet decrease fragmentation.  First, we decided to keep the zero as the first calibrated value because these adolescents do not meet friends during a typical week. This calibration was theoretically important. Second, we decided it was appropriate to have the next calibrated value of ‘sometimes’ meeting friends include the values 1-4. Value 4 is judged to be qualitatively closer to ’sometimes’ than meeting friends very often or daily in all settings. Additionally, a broader range in the middle calibrated value may bring more nuances to the variable. Finally, the calibrated value ‘often’, with values 5-9, implies the adolescent meets friends in two places at least a few days a week and in a third place one day per week or daily in at least one setting and one day per week in the other places, or a few days a week in all settings.  In the aggregated variable of meeting friends online, about half of the adolescents had the maximum value of 6, which was calibrated as the only value included in the highest calibrated value. The value corresponds to daily meeting friends online and through the phone. In contrast, few rated 0-2, and we suspect that very few cases with value 3 responded that they had daily contact with friends using one method, yet no contact using the other method. Therefore, we assigned the lowest calibrated value to include the values 0-3, and the middle value to include values 4-5.  The last question concerning talking to a friend or partner if adolescents are worried, was binary and used as an individual variable. This variable indicates having a close friend or partner to turn to for emotional support. |
|  |  |  |
| **Other leisure-time activities**  **Other leisure-time activities** | How often do you follow the news?  How often do you read other books than schoolbooks?  *In the past 6 months have you been to….?*  *…. a concert?*  *…. the cinema?*  *… a museum?*  *… a library?*  *… the theatre?*  Do you usually attend any other activity *(than sport)* with an adult leader?  How many hours a week do you help with chores at home?  *Have you been drinking alcohol in the past 6 months?*  *Have you been skipping a class in the past 6 months?*  *Have you been smoking in the past 6 months?* | To decrease fragmentation, the multi-value questions of following the news and reading books were calibrated into binary values of not doing this activity or doing it at least weekly.  Having other hobbies may limit the time for physical exercise. To explore which specific activities may be difference-makers for non-participation, these variables were not aggregated.  Doing household chores takes place during a time of the day that may otherwise be allocated to physical exercise and was therefore included in the exploratory phase. The multi-value scale was calibrated to binary, with 2 hours a week or less as the lower value and more than 3 hours a week as the higher.  Drinking alcohol, skipping class, or smoking may correspond to an identity that may be a difference-maker for non-participation in physical exercise. These three variables are, however, different from the other leisure-time activities by their harmful effects on health and rebellious character. The three binary questions were aggregated to a multi-value scale, calibrated into not doing any of these activities or doing one or more. |
|  |  |  |
| **Disability** | Does the adolescent have any of the following: asthma/allergy,  dyslexia,  mobility impairment, ADHD/autism,  hearing impairment, vision impairment not correctable with glasses, or any other disability? | The variable was aggregated before the cluster analysis, on which the outcome is based, and remains aggregated in this study. Breaking it apart and separate it to individual disabilities would increase the fragmentation substantially while not providing any benefit for the interpretation of the results. We assume that adolescents with different types of disabilities experience barriers to participation in physical exercise. Additionally, it is possible to have several types of disabilities, which would further complicate the interpretation of analysis in which disabilities are separated. |

**References**

1. Folkhälsomyndigheten. Utbildning och inkomst som mått på socioekonomi [Internet]. Stockholm: Folkhälsomyndigeten; 2024. [cited 5 May 2025] Available from: https://www.folkhalsomyndigheten.se/publikationer-och-material/publikationsarkiv/u/utbildning-och-inkomst-som-matt-pa-socioekonomi/

2. Lahelma E, Martikainen P, Laaksonen M, Aittomäki A. Pathways between socioeconomic determinants of health. J Epidemiol Community Health. 2004;58(4):327–32.

3. Statistcs Sweden. Socioekonomisk indelning (SEI)[Internet]. Stockholm: Statistics Sweden. [cited 4 May 2025]. Available from: https://www.scb.se/sei

4. Folkhälsomyndigheten. Kunskaper, kompetenser och utbildning – Resultat för uppföljningen av folkhälsopolitikens målområde 2 [Internet]. Stockholm: Folkhälsomyndigeten; 2023. [cited 5 May 2025] Available from: https://www.folkhalsomyndigheten.se/publikationer-och-material/publikationsarkiv/k/kunskaper-kompetenser-och-utbildning-malomrade-2/?pub=120812

5. Rinta-Antila K, Koski P, Heinonen OJ, Korpelainen R, Parkkari J, Savonen K, et al. Educational and family-related determinants of organized sports participation patterns from adolescence to emerging adulthood: A four-year follow-up study. Int J Health Promot Educ. 2022;61(6):317–31.

6. Saunders J, Hume C, Timperio A, Salmon J. Cross-sectional and longitudinal associations between parenting style and adolescent girls’ physical activity. Int J Behav Nutr Phys Act. 2012;9:141.
