# Supplementary material for: Exploring paths to participation and non-participation in physical exercise among Swedish adolescents
Source: Front Public Health. 2026 Feb 3;14:1723898. doi: 10.3389/fpubh.2026.1723898 (PMC12909470; doi:10.3389/fpubh.2026.1723898)
Supplement: Supplementary file 2 [file Table_2.docx]

| **Variable, Question and (numeric) response option** | **(Numeric) Response option** | **Calibration** | **All girls, % (*n*=95)** | **Calibrated, all girls, % (*n*=95)** | **Girls in *Not exercising,* % (*n*=45)** | **Calibrated, girls in *Not exercising,* % (*n*=45)** | **All boys, % (*n*=83)** | **Calibrated, all boys, % (*n*=83)** | **Boys in *Not exercising,* % (*n*=24)** | **Calibrated, boys in *Not exercising,* % (*n*=24)** | |
| --- | --- | --- | --- | --- | --- | --- | --- | --- | --- | --- | --- |
| **Neighborhood** |  |  |  |  |  |  |  |  |  |  | |
| *Are there kids/adolescents you want to avoid in your neighborhood?* | *0: No*  *1: Yes* |  | *No: 87.4 Yes: 12.6* |  | *No: 80.0 Yes: 20.0* |  | *No:78.3 Yes: 20.5 M: 1.2* |  | *No: 79.2 Yes: 20.8* |  | |
| *Are there adults you want to avoid in your neighborhood?* | *0: No*  *1: Yes* |  | *No: 87.4 Yes: 12.6* |  | *No: 84.4 Yes: 15.6* |  | *No: 88.0 Yes: 9.6 M: 2.4* |  | *No: 87.5 Yes: 12.5* |  | |
| **Aggregated variable: Avoiding people in the neighborhood** | **0: Not avoiding 1: Avoiding a few**  **2: Avoiding several** |  | **0:81.0**  **1: 12.6**  **2: 6.3** |  | **0: 75.6 1: 13.3 2: 11.1** |  | **0: 72.3 1: 20.5 2: 3.6 M: 3.6** |  | **0: 70.8 1: 25.0 2: 4.2** |  | |
| *How safe do you feel in your neighborhood during the night?* | *0: very safe*  *1: pretty safe*  *2: not safe* |  | *0: 36.8*  *1: 46.3*  *2: 16.8* |  | *0: 24.4 1: 55.6 2: 20.0* |  | *0: 69.9 1: 26.5 2: 3.6* |  | *0: 70.8 1: 29.2 2: 0* |  | |
| *How safe do you feel in your neighborhood during the day?* | *0: very safe*  *1: pretty safe*  *2: not safe* |  | *0: 77.9*  *1: 22.1*  *2: 0* |  | *0: 73.3 1: 26.7 2: 0* |  | *0: 89.2 1: 10.8 2: 0* |  | *0: 91.7 1: 8.3 2: 0* |  | |
| *How safe do you feel on your way to school?* | *0: very safe*  *1: pretty safe*  *2: not safe* |  | *0: 66.3*  *1: 29.5*  *2: 3.2*  *M: 1.1* |  | *0: 64.4 1: 26.7 2: 6.7 M: 2.2* |  | *0: 88.0 1: 12.0 2: 0* |  | *0: 95.8 1: 4.2 2: 0* |  | |
| **Aggregated variable: Feel safe in the neighborhood** | **0-6** | **0: very safe (0)**  **1: pretty safe (1-2)**  **2: unsafe (3-6)** | 0: 28.4  1: 33.7  2: 20.0  3: 7.4  4: 7.4  5: 2.1  6: 0  M: 1.1 | **0: 28.4**  **1: 53.7**  **2: 16.8**  **M: 1.1** | 0: 15.6 1: 40.0 2: 22.2 3: 11.1 4: 4.4 5: 4.4  6: 0 M: 2.2 | **0: 15.6 1: 62.2 2: 20.0 M: 2.2** | 0: 67.5 1: 18.1 2: 7.2 3: 4.8 4: 2.4 5: 0 6: 0 | **0: 67.5 1: 25.3 2: 7.2** | 0: 70.8 1: 20.8 2: 4.2 3: 4.2  4: 0  5: 0  6: 0 | **0: 70.8 1: 25.0 2: 4.2** | |
|  |  |  |  |  |  |  |  |  |  |  | |
|  |  |  |  |  |  |  |  |  |  |  | |
|  |  |  |  |  |  |  |  |  |  |  | |
|  |  |  |  |  |  |  |  |  |  |  | |
| **Variable, Question and (numeric) response option** | **(Numeric) Response option** | **Calibration** | **All girls, % (n=95)** | **Calibrated, all girls, % (n=95)** | **Girls in *Not exercising,* % (n=45)** | **Calibrated, girls in *Not exercising,* % (*n*=45)** | **All boys, % (n=83)** | **Calibrated, all boys, % (n=83)** | **Boys in *Not exercising,* % (n=24)** | **Calibrated, boys in *Not exercising,* % (n=24)** | |
| **Born in Sweden versus abroad** | | | | | | | | | | |  |
| Country of birth | 0: Born in Sweden, both parents born in Sweden  1: Born in Sweden, one parent born abroad  2: Born in Sweden, both parents born abroad 3: Born abroad | **0: Born in Sweden (0-2)**  **1: Born abroad (3)** | 0: 69.5 1: 6.3  2: 1.1  3: 23.2 | **0: 76.8**  **1: 23.2** | 0: 66.7 1: 0.7 2: 0.7 3: 28.9 | **0: 71.1 1: 28.9** | 0: 73.5 1: 4.8 2: 0 3: 21.7 | **0: 78.3 1: 21.7** | 0: 79.2 1: 4.2 2: 0 3: 16.7 | **0: 83.3 1: 16.6** | |
| **Socioeconomic status** | | | | | | | | | | |  |
| Parents occupation* | 9: Student  11: Not educated, goods producing 12: Not educated, service producing 21: Educated,  goods producing 22: Educated,  service producing 36: Lower official 46: Mid-level official 56-57: Senior official 59: Not classifiable^++^ 60: Independent professional with academic professions 79: Entrepreneur 89: Farmers  99: Unknown^++^ | **1: Student (9)**  **2: Blue collar worker (11, 12, 21, 22)**  **3: White collar worker (36, 46, 56- 57)**  **4: Self-**  **employed (60, 79, 89)**  **Excluded: 59, 99** | 9: 5.3  11: 1.1 12: 2.1 21: 0 22: 4.2 36: 12.6 46: 26.3 56: 28.4 57: 6.3 60: 3.2 79: 8.4 89: 0 99: 2.1 | **1: 5.3**  **2: 7.4**  **3: 73.7**  **4: 11.6**  **M: 2.1** | 9: 6.7  11: 2.2 12: 2.2 21: 0 22: 6.7 36: 6.7  46: 28.9 56: 31.1 57: 0 60: 2.2 79: 8.9 89: 0 99: 4.4 | **1: 6.7 2: 11.1 3: 66.7 4: 11.1 M: 4.4** | 9: 4.8  11: 1.2 12: 6.0 21: 1.2  22: 2.4 36: 7.2  46: 31.3 56: 21.7 57: 6.0 60: 6.0 79: 10.8 89: 0 99: 1.2 | **1: 4.8 2: 10.8 3: 66.3 4: 16.9 M: 1.2** | 9: 4.2  11: 4.2 12: 8.3 21: 0 22: 4.2 36: 0  46: 33.3 56: 20.8 57: 0 60: 8.3 79: 16.6 89: 0 99: 0 | **1: 4.2 2: 16.7 3: 54.2 4: 25.0** | |
|  |  |  |  |  |  |  |  |  |  |  | |
| **Variable, Question and (numeric) response option** | **(Numeric) Response option** | **Calibration** | **All girls, % (n=95)** | **Calibrated, all girls, % (n=95)** | **Girls in *Not exercising,* % (n=45)** | **Calibrated, girls in *Not exercising,* % (*n*=45)** | **All boys, % (n=83)** | **Calibrated, all boys, % (n=83)** | **Boys in *Not exercising,* % (n=24)** | **Calibrated, boys in *Not exercising,* % (n=24)** | |
| If you suddenly need 200 SEK for tomorrow (for example, to go to the movies), could you afford it yourself? | 0: No  1: Yes |  | No: 14.7  Yes: 84.2  M: 1.1 |  | No: 26.7 Yes: 71.1 M: 2.2 |  | No: 7.2 Yes: 92.8 |  | No: 8.3 Yes: 91.7 |  | |
| *During the past six months, has it happened that you have not been able to purchase something you wanted and that many others in your age has, because it was too expensive?* | *0: No*  *1: Once*  *2: Several times* |  | *0: 81.1*  *1: 8.4*  *2: 10.5* |  | *0: 75.6 1: 11.1 2: 13.3* |  | *0: 81.9 1: 13.3 2: 4.8* |  | *0: 70.8 1: 20.8 2: 8.3* |  | |
| *During the past six months, has it happened that you have not been able to join your friends to something because it was too expensive?* | *0: No*  *1: Once*  *2: Several times*  *3: I have no friends ++* |  | *0: 83.2*  *1: 8.4*  *2: 8.4*  *3: 0* |  | *0: 82.2 1: 8.9 2: 8.9*  *3: 0* |  | *0: 94.0 1: 2.4 2: 3.6*  *3: 0* |  | *0: 95.8*  *1: 0*  *2: 4.2*  *3: 0* |  | |
| **Aggregated variable: Money in relation to peers** | **0-4** | **0: equal to peers (0)**  **1: less money than peers (1-4)** | 0: 71.6  1: 8.4  2: 16.8  3: 2.1  4: 1.1 | **0: 71.6**  **1: 28.4** | 0: 64.4 1: 13.3 2: 20.0 3: 0 4: 2.2 | **0: 64.4 1: 35.6** | 0: 79.5 1: 2.4 2: 16.9 3: 1.2 4: 0 | **0: 79.5 1: 20.5** | 0: 70.8 1: 8.3 2: 16.7 3: 4.2 4: 0 | **0: 70.8 1: 29.2** | |
| **School grade** |  |  |  |  |  |  |  |  |  |  | |
| What grade are you in? | 7: Seventh  8: Eight  9: Nine  10: Upper secondary school | **0: Junior secondary school (7-9)**  **1: Upper secondary school (10)** | 7: 3.2  8: 16.8  9: 25.3  10: 52.6  M: 2.1 | **0: 45.3**  **1: 52.6 M: 2.1** | 7: 6.7 8: 17.8 9: 17.8 10: 53.3 M: 4.4 | **0: 42.2 1: 53.3 M: 4.4** | 7: 2.4 8: 19.3 9: 33.7 10: 44.6 | **0: 55.4 1: 44.6** | 7: 4.2 8: 16.7 9: 45.8 10: 33.3 | **0: 66.7 1: 33.3** | |
| **Variable, Question and (numeric) response option** | **(Numeric) Response option** | **Calibration** | **All girls, % (n=95)** | **Calibrated, all girls, % (n=95)** | **Girls in *Not exercising,* % (*n*=45)** | **Calibrated, girls in *Not exercising,* % (*n*=45)** | **All boys, % (n=83)** | **Calibrated, all boys, % (n=83)** | **Boys in *Not exercising,* % (n=24)** | **Calibrated, boys in *Not exercising,* % (n=24)** | |
| **Relationship with mother** | | | | | | | | | | |  |
| *How do you get along with your mom?* | *0: poorly*  *1: pretty poorly*  *2: neither poor nor well*  *3: pretty well*  *4: very well*  *5: I don’t have a mother ++* |  | *0: 0*  *1: 1.1*  *2: 6.3*  *3: 27.4*  *4: 62.1*  *M: 3.2* |  | *0: 0 1: 2.2 2: 4.4 3: 24.4 4: 62.2 M: 6.7* |  | *0: 1.2 1: 0 2: 1.2 3: 25.3 4: 71.1 M: 1.2* |  | *0: 4.2 1: 0 2: 0 3: 33.3 4: 62.5* |  | |
| *Do you talk to your mom if you’re anxious or worried?* | *0: No*  *1: Yes* |  | *No: 32.6*  *Yes: 67.4* |  | *No: 44.4 Yes: 55.6* |  | *No: 16.9 Yes: 81.9 M: 1.2* |  | *No: 20.8 Yes: 79.2* |  | |
| **Aggregated variable: Relationship with mother** | **0-5** | **0: poor (0-2)**  **1: decent (3-4)**  **2: good (5)** | 0: 0  1: 1.1  2: 4.2  3: 14.7  4: 27.4  5: 49.5  M: 3.2 | **0: 5.3**  **1: 42.1**  **2: 49.5 M: 3.2** | 0: 0 1: 2.2 2: 4.4 3: 15.6 4: 26.7 5: 44.4 M: 6.7 | **0: 6.7 1: 42.2 2: 44.4 M: 6.7** | 0: 1.2 1: 0 2: 0 3: 8.4 4: 26.5 5: 62.7 M: 1.2 | **0: 1.2 1: 34.9 2: 62.7 M: 1.2** | 0: 4.2 1: 0 2: 0 3: 4.2 4: 41.7 5: 50.0 | **0: 4.2 1: 45.8 2: 50.0** | |
| **Relationship with father** | | | | | | | | | | |  |
| *How do you get along with your dad?* | *0: poorly*  *1: pretty poorly*  *2: neither poor nor well*  *3: pretty well*  *4: very well*  *5: I don’t have a father ++* |  | *0: 0*  *1: 4.2*  *2: 8.4*  *3: 22.1*  *4: 58.9*  *5: 1.1*  *M: 5.3* |  | *0: 0 1: 4.4 2: 13.3 3: 20.0 4: 53.3 5: 2.2 M: 6.7* |  | *0: 1.2 1: 0 2: 2.4 3: 18.1 4: 71.1 5: 1.2 M: 6.0* |  | *0: 0*  *1: 0*  *2: 4.2*  *3: 25.0*  *4: 62.5 5: 4.2 M: 4.2* |  | |
| *Do you talk to your dad if you’re anxious or worried?* | *0: No*  *1: Yes* |  | *No: 56.8*  *Yes: 43.2* |  | *No: 64.4 Yes: 35.6* |  | *No: 25.3 Yes: 73.5 M: 1.2* |  | *No: 20.8 Yes: 79.2* |  | |
| **Variable, Question and (numeric) response option** | **(Numeric) Response option** | **Calibration** | **All girls, % (n=95)** | **Calibrated, all girls, % (n=95)** | **Girls in *Not exercising,* % (*n*=45)** | **Calibrated, girls in *Not exercising,* % (*n*=45)** | **All boys, % (n=83)** | **Calibrated, all boys, % (n=83)** | **Boys in *Not exercising,* % (n=24)** | **Calibrated, boys in *Not exercising,* % (n=24)** | |
| **Aggregated variable: Relationship with father** | **0-5** | **0: poor (0-2)**  **1: decent (3-4)**  **2: good (5)** | 0: 0  1: 4.2  2: 8.4  3: 18.9  4: 22.1  5: 40.0  M: 6.3 | **0: 12.6**  **1: 41.1**  **2: 40.0 M: 6.3** | 0: 0 1: 4.4 2: 13.3 3: 17.8 4: 22.2 5: 33.3 M: 8.9 | **0: 17.8 1: 40.0 2: 33.3 M: 8.9** | 0: 1.2 1: 0 2: 0 3: 7.2 4: 26.5 5: 57.8 M: 7.2 | **0: 1.2 1: 33.7 2: 57.8 M: 7.2** | 0: 0 1: 0 2: 0 3: 8.3 4: 29.2 5: 54.2 M: 8.3 | **0: 0 1: 37.5 2: 54.2 M: 8.3** | |
| How strict are your parents with knowing where you are if you’re not at home? | 1: very strict  2: pretty strict  3: not that strict  4: not strict at all | **0: not very strict (2-4) 1: very strict (1)** | 1: 49.5  2: 42.1  3: 4.2  4: 3.2  M: 1.1 | **0: 49.5**  **1: 49.5 M: 1.1** | 1: 37.8 2: 48.9 3: 6.7 4: 4.4 M: 2.2 | **0: 60.0 1: 37.8 M: 2.2** | 1: 41.0 2: 50.6 3: 6.0 4: 1.2 M: 1.2 | **0: 57.8 1: 41.0 M: 1.2** | 1: 41.7 2: 54.2 3: 4.2  4: 0 | **0: 58.3 1: 41.7** | |
| **Friendship** | | | | | | | | | | |  |
| *How often do you have friends over?* | 0: Less than weekly  1: One day/week  2: A few days/week  3: Daily |  | *0: 36.8*  *1: 27.4*  *2: 33.7*  *3: 2.1* |  | *0: 46.7 1: 24.4 2: 26.7 3: 2.2* |  | *0: 38.6 1: 30.1 2: 30.1 3: 1.2* |  | *0: 41.7 1: 29.2 2: 29.2 3: 0* |  | |
| *How often are you at a friend’s place?* | 0: Less than weekly  1: One day/week  2: A few days/week  3: Daily |  | *0: 27.4*  *1: 28.4*  *2: 42.1*  *3: 2.1* |  | *0: 46.7 1: 20.0 2: 31.1 0: 2.2* |  | *0: 25.3 1: 25.3 2: 45.8 3: 3.6* |  | *0: 37.5 1: 29.2 2: 29.2 3: 4.2* |  | |
| *How often do you meet friends elsewhere than at someone’s home?* | 0: Less than weekly  1: One day/week  2: A few days/week  3: Daily |  | *0: 14.7*  *1: 18.9*  *2: 47.4*  *3: 18.9* |  | *0: 22.2 1: 20.0 2: 46.7 3: 11.1* |  | *0: 9.6 1: 12.0 2: 53.0 3: 25.3* |  | *0: 12.5 1: 25.0 2: 41.7 3: 20.8* |  | |
| **Aggregated variable: Meeting friends physically** | **0-9** | **0: Less than weekly (0)**  **1: Weekly (1-4)**  **2: More often (5-9)** | 0: 5.3  1: 5.3  2: 12.6  3: 14.7  4: 23.2  5: 17.9  6: 13.7  7: 7.4  8: 0  9: 0 | **0: 5.3**  **1: 55.8**  **2: 38.9** | 0: 8.9 1: 11.1 2: 20.0 3: 20.0 4: 13.3 5: 6.7 6: 17.8 7: 2.2 8: 0 9: 0 | **0: 8.9 1: 64.4 2: 26.7** | 0: 3.6 1: 2.4 2: 19.3 3: 9.6 4: 24.1 5: 12.0 6: 16.8 7: 8.4 8: 3.6 9: 0 | **0: 3.6 1: 55.4 2: 41.0** | 0: 8.3 1: 4.2 2: 16.7 3: 25.0 4: 20.8 5: 4.2 6: 8.3 7: 8.3 8: 4.2 9: 0 | **0: 8.3 1: 66.7 2: 25.0** | |
| **Variable, Question and (numeric) response option** | **(Numeric) Response option** | **Calibration** | **All girls, % (n=95)** | **Calibrated, all girls, % (n=95)** | **Girls in *Not exercising,* % (*n*=45)** | **Calibrated, girls in *Not exercising,* % (*n*=45)** | **All boys, % (n=83)** | **Calibrated, all boys, % (n=83)** | **Boys in *Not exercising,* % (n=24)** | **Calibrated, boys in *Not exercising,* % (n=24)** | |
| *How often do you meet friends online?* | 0: Less than weekly  1: One day/week  2: A few days/week  3: Daily |  | *0: 7.4*  *1: 3.2*  *2: 11.6*  *3: 77.9* |  | *0: 11.1 1: 4.4 2: 11.1 3: 73.3* |  | *0: 6.0 1: 6.0 2: 26.5 3: 61.4* |  | *0: 12.5 1: 8.3 2: 29.2 3: 50.0* |  | |
| *How often do you talk on the phone or text message with friends?* | 0: Less than weekly  1: One day/week  2: A few days/week  3: Daily |  | *0: 6.3*  *1: 2.1*  *2: 20.0*  *3: 63.2* |  | *0: 6.7 1: 4.4 2: 20.0 3: 68.9* |  | *0: 4.8 1: 4.8 2: 12.0 3: 78.3* |  | *0: 4.2 1: 8.3 2: 12.5 3: 75.0* |  | |
| **Aggregated variable: Meeting friends digitally** | **0-6** | **0: Rarely (0-3)**  **1: Often (4-5)**  **2: Daily (6)** | 0: 1.1  1: 1.1  2: 2.2  3: 10.5  4: 6.4  5: 18.9  6: 60.0 | **0: 14.7**  **1: 25.3**  **2: 60.0** | 0: 0 1: 2.2 2: 2.2 3: 17.8 4: 6.7 5: 15.6 6: 55.6 | **0: 22.2 1: 22.2 2: 55.6** | 0: 0 1: 0 2: 4.8 3: 9.6 4: 10.8 5: 22.9 6: 51.8 | **0: 14.5 1: 33.7 2: 51.8** | 0: 0 1: 0 2: 8.3 3: 12.5 4: 16.7 5: 20.8 6: 41.7 | **0: 20.8 1: 37.5 2: 41.7** | |
| If you worry about something, do you talk to your partner/friend? | 0: No  1: Yes |  | No: 36.8  Yes: 63.2 |  | No: 37.8 Yes: 62.2 |  | No: 48.2 Yes: 50.6 M: 1.2 |  | No: 62.5  Yes: 37.5 |  | |
| **Other leisure-time activities** | | | | | | | | | | |  |
| How often do you follow the news? | 0: Less than weekly  1: One day/week  2: A few days/week  3: Daily | **0: Never (0)**  **1: Weekly or more often (1-3)** | 0: 15.8  1: 11.6  2: 47.4  3: 25.3 | **0: 15.8**  **1: 84.2** | 0: 28.9 1: 11.1 2: 37.8 3: 22.2 | **0: 28.9 1: 71.1** | 0: 14.5 1: 14.5 2: 44.6 3: 26.5 | **0: 14.5 1: 85.5** | 0: 20.8 1: 20.8 2: 41.7 3: 16.7 | **0: 20.8 1: 79.2** | |
| How often do you read other books than schoolbooks? | 0: Less than weekly  1: One day/week  2: A few days/week  3: Daily | **0: Never (0)**  **1: Weekly or more often (1-3)** | 0: 53.7  1: 15.8  2: 20.0  3: 10.5 | **0: 53.7**  **1: 46.3** | 0: 62.2 1: 4.4 2: 20.0 3: 13.3 | **0: 62.2 1: 37.8** | 0: 60.2 1: 14.5 2: 19.3 3: 6.0 | **0: 60.2 1: 39.8** | 0: 79.2 1: 16.7 2: 0  3: 4.2 | **0: 79.2 1: 20.8** | |
| Have you been to a concert in the past 6 months? | 0: No  1: Yes |  | No: 58.9  Yes: 41.1 |  | No: 68.9 Yes: 31.1 |  | No: 73.5 Yes: 26.5 |  | No: 75.0 Yes: 25.0 |  | |
| **Variable, Question and (numeric) response option** | **(Numeric) Response option** | **Calibration** | **All girls, % (n=95)** | **Calibrated, all girls, % (n=95)** | **Girls in *Not exercising,* % (*n*=45)** | **Calibrated, girls in *Not exercising,* % (*n*=45)** | **All boys, % (n=83)** | **Calibrated, all boys, % (n=83)** | **Boys in *Not exercising,* % (n=24)** | **Calibrated, boys in *Not exercising,* % (n=24)** | |
| Have you been to the cinema in the past 6 months? | 0: No  1: Yes |  | No: 21.1  Yes: 78.9 |  | No: 31.1 Yes: 68.9 |  | No: 19.3 Yes: 79.5 M: 1.2 |  | No: 20.8 Yes: 79.2 |  | |
| Have you been to a museum in the past 6 months? | 0: No  1: Yes |  | No: 72.6  Yes: 26.3  M: 1.1 |  | No: 75.6 Yes: 24.4 |  | No: 69.9 Yes: 30.1 |  | No: 79.2 Yes: 20.8 |  | |
| Have you been to a library in the past 6 months? | 0: No  1: Yes |  | No: 34.7  Yes: 65.3 |  | No: 33.3 Yes: 66.7 |  | No: 47.0 Yes: 53.0 |  | No: 54.2 Yes: 45.8 |  | |
| Have you been to the theatre in the past 6 months? | 0: No  1: Yes |  | No: 76.8  Yes: 23.2 |  | No: 88.9 Yes: 11.1 |  | No: 80.7 Yes: 19.3 |  | No: 91.7 Yes: 8.3 |  | |
| Do you usually attend any other activity (than sport) with an adult leader? | 0: No  1: Yes |  | No: 89.5  Yes: 10.5 |  | No: 93.3 Yes: 6.7 |  | No: 85.5 Yes: 14.5 |  | No: 62.5 Yes: 37.5 |  | |
| How many hours a week do you help with chores at home? | 1: less than 1 hour  2: 1-2 hours  3: 3-4 hours  4: 5 hours or more | **0: 0-2 hours (1-2)**  **1: More than 3 hours (3-4)** | 1: 15.8  2: 56.8  3: 21.1  4: 6.3 | **0: 72.6**  **1: 27.4** | 1: 15.6 2: 66.7 3: 11.1 4: 6.7 | **0: 82.2 1: 17.8** | 1: 8.4 2: 59.0 3: 22.9 4: 7.2 M: 2.4 | **0: 67.5 1: 30.1 M: 2.4** | 1: 16.7 2: 54.2 3: 25.0 4: 4.2 | **0: 70.8 1: 29.2** | |
| *Have you been drinking alcohol in the past 6 months?* | *0: No*  *1: Yes* |  | *No: 70.5*  *Yes: 29.5* |  | *No: 77.8 Yes: 22.2* |  | *No: 68.7 Yes: 31.3* |  | *No: 62.5 Yes: 37.5* |  | |
| *Have you been skipping a class in the past 6 months?* | *0: No*  *1: Yes* |  | *No: 82.1*  *Yes: 16.8*  *M: 1.1* |  | *No: 75.6 Yes: 22.2 M: 2.2* |  | *No: 84.3 Yes: 15.7* |  | *No: 87.5 Yes: 12.5* |  | |
| *Have you been smoking in the past 6 months?* | *0: No*  *1: Yes* |  | *No: 89.5*  *Yes: 10.5* |  | *No: 93.3 Yes: 6.7* |  | *No: 92.8 Yes: 7.2* |  | *No: 91.7 Yes: 8.3* |  | |
| **Variable, Question and (numeric) response option** | **(Numeric) Response option** | **Calibration** | **All girls, % (n=95)** | **Calibrated, all girls, % (n=95)** | **Girls in *Not exercising,* % (*n*=45)** | **Calibrated, girls in *Not exercising,* % (*n*=45)** | **All boys, % (n=83)** | **Calibrated, all boys, % (n=83)** | **Boys in *Not exercising,* % (n=24)** | **Calibrated, boys in *Not exercising,* % (n=24)** | |
| **Aggregated variable: Harmful behaviors** | **0-3** | **0: no bad behavior (0)**  **1: one or more (1-3)** | 0: 65.3  1: 18.9  2: 7.4  3: 7.4  M: 1.1 | **0: 65.3**  **1: 33.7 M: 1.1** | 0: 66.7 1: 15.6 2: 13.3 3: 2.2 M: 2.2 | **0: 66.7 1: 31.1 M: 2.2** | 0: 59.0 1: 28.9 2: 10.8 3: 1.2 | **0: 59.0 1: 41.0** | 0: 54.2 1: 37.5 2: 4.2 3: 4.2 | **0: 54.2 1: 45.8** | |
| **Disability*** |  |  |  |  |  |  |  |  |  |  | |
| Does the adolescent have any of the following: asthma/allergy, dyslexia,  mobility impairment, ADHD/autism,  hearing impairment, vision impairment not correctable with glasses,  or any other disability? | 0: No  1: Yes |  | No: 81.1  Yes: 18.9 |  | No: 82.2 Yes: 17.8 |  | No: 74.7 Yes: 25.3 |  | No: 62.5 Yes: 37.5 |  | |
